# Caregiver self-efficacy providing nutritional support for pediatric patients undergoing hematopoietic stem cell transplant is associated with psychosocial factors

**DOI:** 10.3389/fnut.2024.1323482

**Published:** 2024-02-29

**Authors:** Leah LaLonde, Alexandra Neenan, Michelle Byrd, Flora Hoodin, Sandra Bouma, Sung Won Choi

**Affiliations:** ^1^Department of Psychology, Eastern Michigan University, Ypsilanti, MI, United States; ^2^Department of Psychology, Nationwide Children's Hospital, Columbus, OH, United States; ^3^Department of Psychiatry, University of Michigan, Ann Arbor, MI, United States; ^4^Department of Pediatrics, University of Michigan, Ann Arbor, MI, United States; ^5^Rogel Comprehensive Cancer Center, University of Michigan, Ann Arbor, MI, United States

**Keywords:** nutrition, caregivers, HSCT, supportive care, dietary self-efficacy

## Abstract

**Introduction:**

Caregiver self-efficacy in providing nutritional support to pediatric hematopoietic stem cell transplantation (HSCT) patients has been little studied despite the increased risk of these children potentially being over- or under-nourished after HSCT, and nutritional status could possibly affect treatment outcomes. The current study aimed to describe caregiver dietary self-efficacy and its associated psychosocial factors and barriers to following dietary recommendations.

**Methods:**

Caregivers completed questionnaires pre-HSCT and 30 days, 100 days, and one year post-HSCT. A subset provided a 24-h recall of food intake.

**Results:**

Results showed generally high caregiver confidence and low difficulty supporting their child nutritionally. However, lower confidence was associated with higher caregiver depression, anxiety, and stress 30 days post-HSCT. Further, higher difficulty at various time points was correlated with lower income, higher depression and anxiety, stress, and miscarried helping (i.e., negative caregiver-child interactions surrounding eating), as well as child overweight status and failure to meet protein intake guidelines. Nutritional criteria for protein, fiber, added sugar, and saturated fat were met by 65%, 0%, 75%, and 75%, respectively. Caregiver attitudes and child behavior were the most frequently reported barriers to healthy eating.

**Discussion:**

Results suggest that directing resources to caregivers struggling emotionally, economically, or transactionally could support pediatric patients undergoing HSCT in maintaining optimal nutritional status.

## 1 Introduction

Children undergoing hematopoietic stem cell transplant (HSCT) are required to follow strict medical regimens, including taking medication, deploying behavioral strategies to minimize infections, and following dietary guidelines. Adhering to these dietary guidelines can be particularly challenging as pre-HSCT conditioning regimens can adversely affect their food intake (1), much like infections, various medications, and the well-described post-HSCT complication, graft-vs.-host disease (2, 3).

Few studies have investigated child nutritional status prior to, during, and after HSCT. One such investigation of pediatric HSCT survivors (median time since transplant 636 days) revealed that nearly one-quarter of pediatric patients were under-nourished, and 28% were over-nourished (4). Poor nutritional status has the potential to negatively influence medical outcomes, such as lowered chemotherapy tolerance, altered metabolism of drugs, and increased infections due to more immunosuppression (5). A recent meta-analysis reported that lower than normal body mass index (BMI) before or during HSCT was significantly associated with poorer overall survival and poorer event-free survival compared to patients with BMI within the healthy range (6). In contrast, in single-center studies, pre-HSCT underweight status was not associated with mortality risk, whereas overweight status was associated with poorer outcomes following allogeneic- or autologous-HSCT (7). Patients who were overweight or obese 100 days post-HSCT had poorer 5-year overall survival (8). However, a retrospective analysis of 3,687 children found that pre-allogeneic HSCT BMI was not significantly associated with survival (9). Thus, the literature has no consensus as yet regarding the effect of pre-HSCT BMI on outcomes post-HSCT.

Psychosocial factors that impact child eating behavior and nutritional status have seldom been examined among families of pediatric patients undergoing HSCT. In particular, the mental health and distress of caregivers who are responsible for overseeing the nutritional intake have received little attention. Studies in non-cancer samples show early maternal depression predicts subsequent child food responsiveness, which in turn predicts higher BMI (10). Further, maternal symptoms of anxiety and depression are associated with less frequent monitoring of child feeding and more relinquishing of control over food consumption to the children (11). Stressed mothers are also less likely to engage in proactive healthy meal planning and more likely to provide children with food high in fat and sugar (12, 13). An additional transactional dynamic is that parental efforts to help improve their child's nutritional status may miscarry or lead to unintended consequences, such as negative parent-child health-related interactions, which then may contribute to poorer child outcomes (“miscarried helping”) (14).

In the context of cancer caregiving, one study found a child's reluctance to eat secondary to treatment side effects is a common source of stress in caregivers of pediatric cancer patients (1). Caregiver stress may contribute to mealtime transactions that inadvertently undermine parental efforts to facilitate child adherence to eating recommended foods. The more demanding the parental feeding style, the more problematic the child's eating behavior and food refusal (15). Parental stress may be moderated by caregiver self-efficacy (i.e., perceived ability to meet the demands of caregiving) or by caregiver activation, which involves knowledge, skill, confidence, and motivation to play an active role in health care (16) and thus managing their child's health care and by extension, their child's eating. In turn, caregiver self-efficacy and activation in facilitating child adherence to dietary guidelines is likely influenced by multiple factors, including perceived difficulties, barriers to care, the parent-patient dyadic interaction, perceived benefits of dietary guidelines, perceived seriousness of leaving the diet unaddressed, workable plans, financial resources, and opportunity to adhere [see Health Belief Model (17) and Theory of Planned Behavior (18)].

If the nutritional status of the child is viewed as a prognostic factor of treatment outcome, then supporting their caregiver's nutritional support behavior may be an important avenue to improving the child's nutritional status and treatment outcomes. Hence, the primary objective of the current study is to characterize caregiver self-efficacy in their role of supporting child nutrition in pediatric patients undergoing HSCT and catalog caregivers' perceived barriers to the child's healthy eating behaviors. The secondary objective is to identify how the caregivers' self-efficacy in providing nutritional support to their child may be associated with (1) caregiver factors, such as stress, depression, and anxiety; (2) child and caregiver interactions including miscarried helping; and (3) child nutritional status, including BMI and nutritional intake.

## 2 Materials and methods

### 2.1 Participants

Caregivers were recruited from a Midwest academic health center between March 2016 and February 2020. Inclusion criteria specified participants be proficient in English and primary caregivers of pediatric HSCT candidates, ages 1–22 years; patients up to age 25 years receive transplants in the Pediatric HSCT Unit at the center. After consenting to participate, caregivers completed questionnaires on an iPad during their child's clinic appointments pre-HSCT and 30 days, 100 days, and 1 year post-HSCT.

### 2.2 Measures

Medical and anthropometric patient data of patients extracted from the electronic health record included medical diagnosis, type of transplant (autologous or “self-donor” vs. allogeneic or “other than self-donor”), height and height for age Z-score, weight and weight for age Z-score, and BMI and BMI Z-score. The Z-scores allow for comparisons across age and sex and are useful for assessing longitudinal changes and help identify children with extreme values (19).

Caregivers completed the following questionnaires:

The demographics questionnaire was tailored to this study and gathered caregiver and patient demographics, including caregiver relationship to child, race/ethnicity, sex, education, relationship status, employment status, yearly household income, and patient age and race/ethnicity.

The Dietary Self-Efficacy questionnaire assessed caregiver self-efficacy for optimally supporting their child's nutrition. In the absence of caregiving dietary self-efficacy scales for children undergoing HSCT, this questionnaire was developed specifically for this study through multi-disciplinary collaboration with hematology/oncology, dietetics, and psychology. The questionnaire was based on variables identified within the adherence and health behaviors literature (18, 20).

*Knowledge:* To assess dietary self-efficacy, caregivers first rated their knowledge of what types of foods are healthy for their child undergoing HSCT. Each item was rated on a 5-point scale ranging from 0 (very unhealthy) to 4 (very healthy). For purposes of our analyses, food types were further categorized into nutrient-dense foods or calorie-dense foods.
*Importance:* Caregivers also rated their perceptions of how important it is that their child consumes those same types of foods from 0 (not at all important) to 4 (extremely important).
*Confidence:* On a scale from 0 (not at all) to 4 (extremely), caregivers rated their confidence in their knowledge of what food is healthy in general, what food is healthy for the child undergoing HSCT, their ability to gather and prepare healthy foods, and to teach the child about healthy eating.
*Perceived difficulty:* Caregivers reported perceived difficulty gathering, preparing, and affording healthy foods on the same scale from 0 (not at all) to 4 (extremely).
*Barriers:* Caregivers checked which items on a list of 22 potential barriers they perceived might get in the way of providing healthy foods for the child undergoing HSCT.

The Patient Health Questionnaire-4 (PHQ-4) (21, 22) assessed caregiver symptoms of anxiety and depression, which are often comorbid. The PHQ-4 is a 4-item ultra-brief screening measure. Total scores range from 0 to 12, with higher scores indicating a higher symptom burden of anxiety and depression.

The Perceived Stress Scale (PSS) (23) assessed general levels of caregiver stress. The PSS is a 10-item measure assessing perceived unpredictability, uncontrollability, and overload of stressors faced during the last month. Each item was rated using a Likert scale from 0 (never) to 4 (always). Total scores range from 0 to 40, with higher scores indicating greater stress.

The Helping for Health Inventory (HHI) (24) assessed caregiver-child interaction surrounding eating. The HHI is a 15-item caregiver report measure assessing miscarried helping, which is the degree to which caregivers' well-intentioned efforts become barriers to the management of youth chronic illness. As the scale was initially developed in a diabetes sample, we replaced the word “diabetes” with “eating” for the purpose of the current study. Caregivers rated items on a scale from 1 (rarely) to 5 (always). Total scores range from 15 to 75, with higher scores indicating more miscarried helping. Internal consistency in the current sample was not adequate (Cronbach's alpha = 0.46), although prior studies found the HHI to be internally reliable in diabetes samples (24).

To assess food and nutrient intake, patients and/or their caregivers completed an age-appropriate, online, and validated food and activity questionnaire [BLOCK by Nutrition Quest (25, 26)] using a laptop computer at the end of their clinic visit. The patient report was used for participants 16 and older using the BLOCK Kids food and activity questionnaire, which is designed to assess usual dietary intake over the past 7 days; patients who were 18 and older used the BLOCK Alive! food and activity questionnaire. The caregiver report was used for patients between ages 3 and 16. However, one caregiver of a 17-year-old participant completed the BLOCK Kids food frequency questionnaire on behalf of the teenaged patient. Participant data were downloaded from the password-protected Nutrition Quest research portal for analysis. From these reports, four food intake composites were created: fiber (from fruit, vegetable, and whole grain), protein, added sugar, and saturated fat intake. Dichotomous variables were created based on whether the patient met dietary guidelines for their age and gender, with higher intakes of fiber and protein and lower intakes of added sugar and saturated fat indicating a healthier diet (27).

### 2.3 Statistical analysis

Continuous measures were summarized by sample mean (M) and standard deviation (SD); categorical measures were summarized with frequency counts and percentages of the sample in each category. *T*-tests were used to compare healthiness ratings and importance ratings of calorie-dense foods and nutrient-dense foods at each time point and differences in caregiver-rated importance of diet compared to other health behaviors. One-Way ANOVAs were utilized to assess differences in healthiness and importance ratings of calorie-dense foods and nutrient-dense foods across pre-HSCT, day 30, day 100, and 1 year post-HSCT. One-Way ANOVAs were also used to assess differences in confidence and difficulty in providing healthy food to their child across time. Pearson's, Spearman's, and point biserial correlations were utilized to assess associations between dietary self-efficacy, caregiver-reported barriers, child and caregiver factors, and caregiver-child interactions.

Child weight and height were used to calculate body mass index (BMI) using the formula: weight in kilograms divided by height in meters squared for all participants aged 20 years and older. For participants between the ages of 2 and 19 years, BMI Z-scores were utilized. Independent sample *t*-tests were used to assess differences in dietary self-efficacy, caregiver factors, and caregiver-child interactions between caregivers of pediatric patients who met nutritional guidelines for protein, sugar, and fat and caregivers of pediatric patients who did not meet nutritional guidelines. Pearson's correlations were used to assess the association between fiber intake and dietary self-efficacy, caregiver factors, and caregiver-child interactions. One-Way ANOVAs were used to assess differences in dietary self-efficacy among caregivers of pediatric patients who fell within the underweight, healthy, or overweight ranges. Statistical significance was defined as a *p* < 0.05. Analyses were performed using SPSS (version 28).

## 3 Results

### 3.1 Descriptive statistics

Prior to HSCT, 46 caregivers of pediatric patients aged 1–22 (M = 10.33, SD = 6.86) completed the questionnaires. The most common pediatric diagnoses were neuroblastoma (*n* = 13), Acute Lymphocytic Leukemia (ALL) (*n* = 10), Acute Myeloid Leukemia (AML) (*n* = 5), acquired severe aplastic anemia (*n* = 4), and Hodgkin's lymphoma (*n* = 3). Other diagnoses included Kostmann syndrome (*n* = 2), acute biphenotypic leukemia (*n* = 1), Central Nervous System (CNS) tumor (*n* = 1), chronic granulomatous disease (*n* = 1), Ewing's sarcoma (*n* = 1), GATA haploinsufficiency (*n* = 1), hemophagocytic lymphohistiocytosis (*n* = 1), juvenile myelomonocytic leukemia (*n* = 1), NFKBIA Mutation (*n* = 1), and sickle cell disease (*n* = 1).

A total of 27 pediatric patients underwent allogeneic transplants, and 18 underwent autologous transplants. One pediatric patient and caregiver who completed only pre-HSCT data did not return for the transplant. A total of 32 caregivers completed questionnaires at day 30, 31 caregivers at day 100, and 14 caregivers at 1 year post-HSCT. Caregivers who dropped out of the study did not significantly differ from caregivers who remained in the study at day 30, day 100, and 1 year post-HSCT. Caregivers were primarily female, white, married, and completed, on average, some college education or an Associate's degree (Table 1).

**Table 1:** Caregiver and pediatric patient demographics (*n* = 46).

	**Caregiver**		**Child**	
	***n***	**%**	***n***	**%**
**Gender**				
Female	43	(94)	14	(30)
Male	3	(6)	32	(70)
**Race/ethnicity**				
White	30	(65)	29	(64)
Black/African American	10	(22)	9	(20)
Hispanic/Latinx	2	(4)	2	(4)
Asian	2	(4)	1	(2)
Middle Eastern	1	(2)	1	(2)
Mixed	1	(2)	3	(7)
**Relationship status**				
Married	27	(56)		
Single	9	(20)		
Divorced	5	(5)		
Separated	1	(2)		
Living with Partner	4	(9)		
**Education**				
Less than high school diploma	3	(7)		
High school diploma or equivalent	10	(22)		
Some college/Associate's	16	(35)		
Bachelor's degree	7	(15)		
Master's degree	4	(9)		
Other	6	(13)		
**Annual household income**				
< $9,000	3	(7)		
$10,000–$24,000	7	(15)		
$25,000–$49,000	8	(17)		
$50,000–$74,000	4	(9)		
$75,000–$99,000	7	(15)		
$100,000–$149,000	6	(13)		
>$150,000	5	(11)		
Chose not to disclose	6	(13)		

Demographic information collected at the pre-HSCT time point only.

Caregiver depression and anxiety were highest pre-HSCT when ~13% of caregivers fell within the moderate to severe distress range, 20% fell within the mild distress range, and 67% fell within the average or not clinically elevated distress range. At day 30 or day 100 post-HSCT, none of the caregivers fell within the moderate or severe distress range. At one year post-HSCT, one caregiver's distress level fell within the moderate severity range (Table 2). Child age was positively correlated with caregiver distress only at day 30 post-HSCT (*r* = 0.35, *p* < 0.05).

**Table 2:** Caregiver distress descriptive statistics.

	**Time point**	***n***	**%**	**Mean**	**SD**	**Range**
PHQ-4	Baseline	46		2.15	2.40	0–9
	Normal	31	(67)			0–2
	Mild	9	(20)			3–5
	Moderate	5	(11)			6–8
	Severe	1	(2)			9 −12
	Day 30	32		1.43	1.64	0–5
	Normal	22	(69)			0–2
	Mild	10	(31)			3–5
	Day 100	31		1.73	1.79	0–5
	Normal	20	(65)			0–2
	Mild	10	(32)			3–5
	1 Year	14		1.28	2.27	0–8
	Normal	12	(86)			0–2
	Mild	1	(7)			3–5
	Moderate	1	(7)			6–8
HHI	Baseline	46		28.83	8.13	3–61
	Day 30	31		30.58	8.20	14–56
	Day 100	32		29.61	10.50	9–54
	1 Year	14		27.57	6.24	15–35
PSS	Baseline	46		18.50	5.74	0–29
	Day 30	32		18.00	5.35	0–28
	Day 100	31		17.80	3.55	10–23
	1 Year	14		15.86	3.41	6–20

n, sample size; SD, standard deviation; Range is the observed range reported in the sample. PHQ−4, Patient Health Questionnaire 4–items; HHI, Helping for Health Inventory; PSS, Perceived Stress Scale. Higher scores represent greater distress (PHQ-4), miscarried helping (HHI), and stress (PSS). One missing PHQ-4 and PSS data point at day 100 post-HSCT.

A subset of the participants completed the Nutrition Quest BLOCK food frequency questionnaire. A total of 19 caregivers or pediatric patients completed Nutrition Quest pre-HSCT; 11 completed Nutrition Quest on day 30, 9 on day 100, and 4 on 1 year post-HSCT.

### 3.2 Dietary self-efficacy

#### 3.2.1 Knowledge

**Figure 1:**
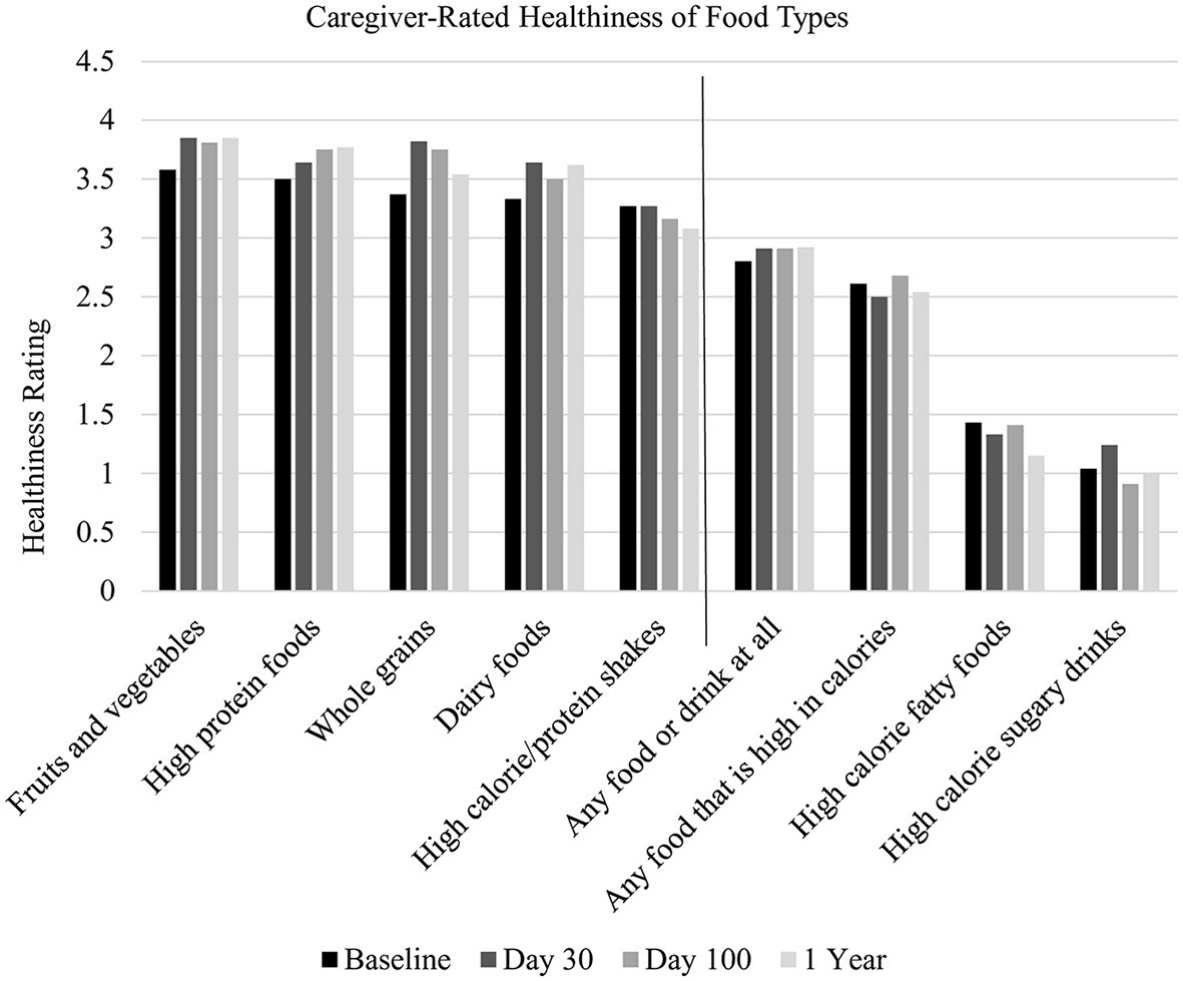
Caregiver-rated healthiness of food types. Nutrient-dense foods are displayed to the left of the vertical line, and calorie-dense foods are displayed to the right. X-axis labels: 0 = Very unhealthy, 1 = Unhealthy, 2 = Somewhat healthy, 3 = Healthy, and 4 = Very healthy.

Overall, caregivers reported nutrient-dense foods (i.e., high protein, fruits, vegetables, and grains) as *somewhat healthy* or *healthy* and calorie-dense foods (i.e., any food or drink including high sugar and high fat) as *unhealthy* (Figure 1). At all-time points, caregivers rated nutrient-dense foods as significantly healthier than calorie-dense foods (Table 3). Caregiver healthiness ratings of nutrient-dense foods did not significantly change over time [*F*_(3, 119)_ = 2.12, *p* = 0.10], nor did their healthiness rating of calorie-dense foods [*F*_(3, 119)_ = 0.01, *p* = 0.99].

**Table 3:** Knowledge and importance of nutrient-dense and calorie-dense foods.

	**Time point**		**Mean**	**SD**	**Sig**.
Knowledge^a^	Pre-HSCT	Nutrient-dense	3.38	0.62	
		Calorie-dense	1.97	0.88	^**^
	Day 30	Nutrient-dense	3.64	0.39	
		Calorie-dense	1.98	0.92	^**^
	Day 100	Nutrient-dense	3.59	0.45	
		Calorie-dense	1.95	0.82	^**^
	1 Year	Nutrient-dense	3.57	0.48	
		Calorie-dense	1.90	1.18	^**^
Importance^b^	Pre-HSCT	Nutrient-dense	3.14	0.65	
		Calorie-dense	2.88	0.65	0.06
	Day 30	Nutrient-dense	3.16	0.54	
		Calorie-dense	2.78	0.67	^*^
	Day 100	Nutrient-dense	3.19	0.53	
		Calorie-dense	2.60	0.79	^**^
	1 Year	Nutrient-dense	3.08	0.65	
		Calorie-dense	2.33	0.81	^*^

^*^p < 0.05, ^**^p < 0.01. ^a^Higher scores = higher rated healthiness of calorie-dense or nutrient-dense foods. ^b^Higher scores = higher rated importance of nutrient-dense or calorie-dense foods. Nutrient-dense foods = Protein, fruits and vegetables, grains, and dairy. Calorie-dense foods = Sugar, fat, and any high calorie. Sig = statistical significance of t tests for dependent means comparing (a) knowledge of healthiness of calorie-dense vs. nutrient-dense foods and (b) importance of nutrient-dense vs. calorie-dense foods.

Pre-HSCT, caregiver healthiness ratings of calorie-dense foods and child age were significantly correlated (*r* = −0.36, *p* = 0.05), such that older child age was associated with lower rated healthiness of calorie-dense foods. At no other time point were caregiver ratings of healthiness of calorie-dense foods significantly correlated with child age. Similarly, the caregiver's healthiness rating of nutrient-dense foods was not significantly correlated with the child's age at any time point.

#### 3.2.2 Importance

**Figure 2:**
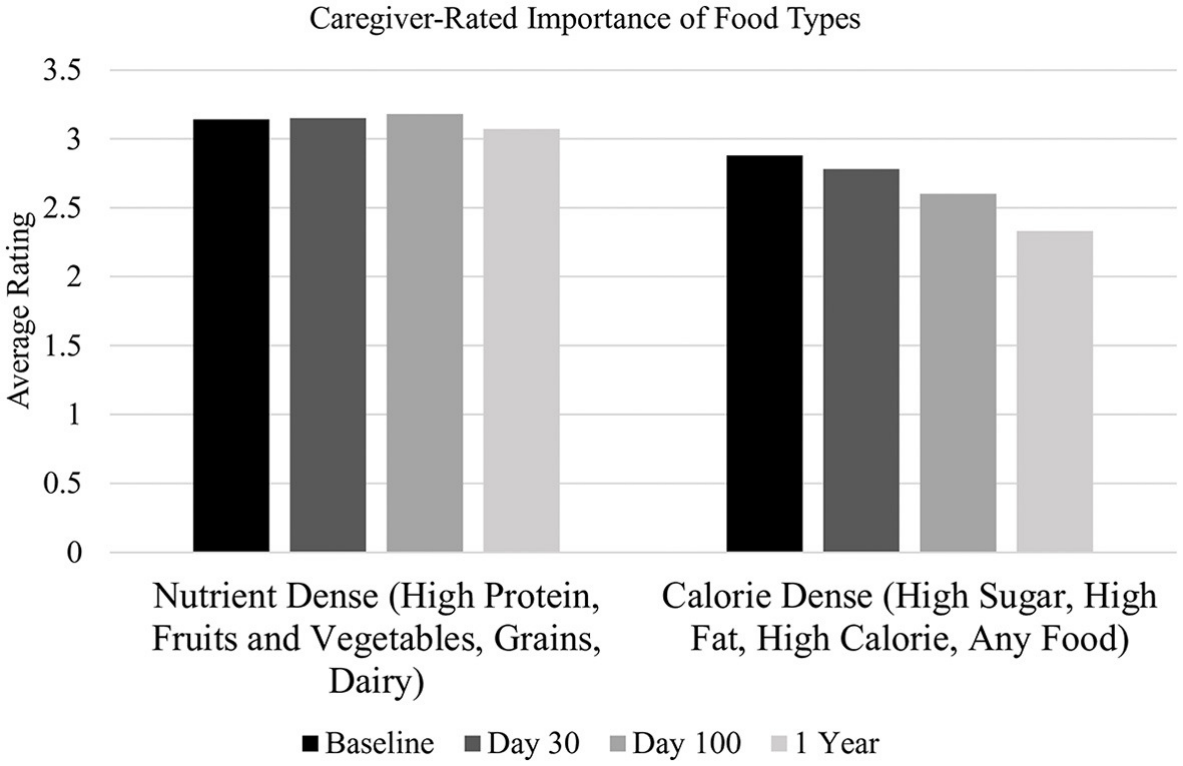
Caregiver-reported importance of food types. X-axis labels: 0 = not at all important, 1 = Unimportant, 2 = Somewhat important, 3 = Important, and 4 = Extremely important.

Overall, caregivers rated nutrient-dense foods as *important* for their child (see Figure 2) and rated calorie-dense foods *as somewhat important*. The caregiver-rated importance of nutrient-dense foods [*F*_(3, 119)_ = 0.09, *p* = 0.96] and calorie-dense foods did not significantly change [*F*_(3, 119)_ = 2.29, *p* = 0.08] across time. Pre-HSCT, caregiver-rated importance of nutrient-dense and calorie-dense foods did not significantly differ. At all remaining time points, caregivers rated nutrient-dense foods as significantly more important than calorie-dense foods (Table 3).

Caregiver-rated importance of calorie-dense foods was significantly correlated with child age at day 30 (*r* = −0.36, *p* < 0.05) and day 100 (*r* = −0.40, *p* < 0.05), such that younger child age was associated with caregiver rating of calorie-dense foods as more important. Pre-HSCT and one year post-HSCT, caregiver-rated importance of calorie-dense foods was not significantly correlated with child age. At no time point was caregiver-rated importance of nutrient-dense foods significantly correlated with child age.

**Figure 3:**
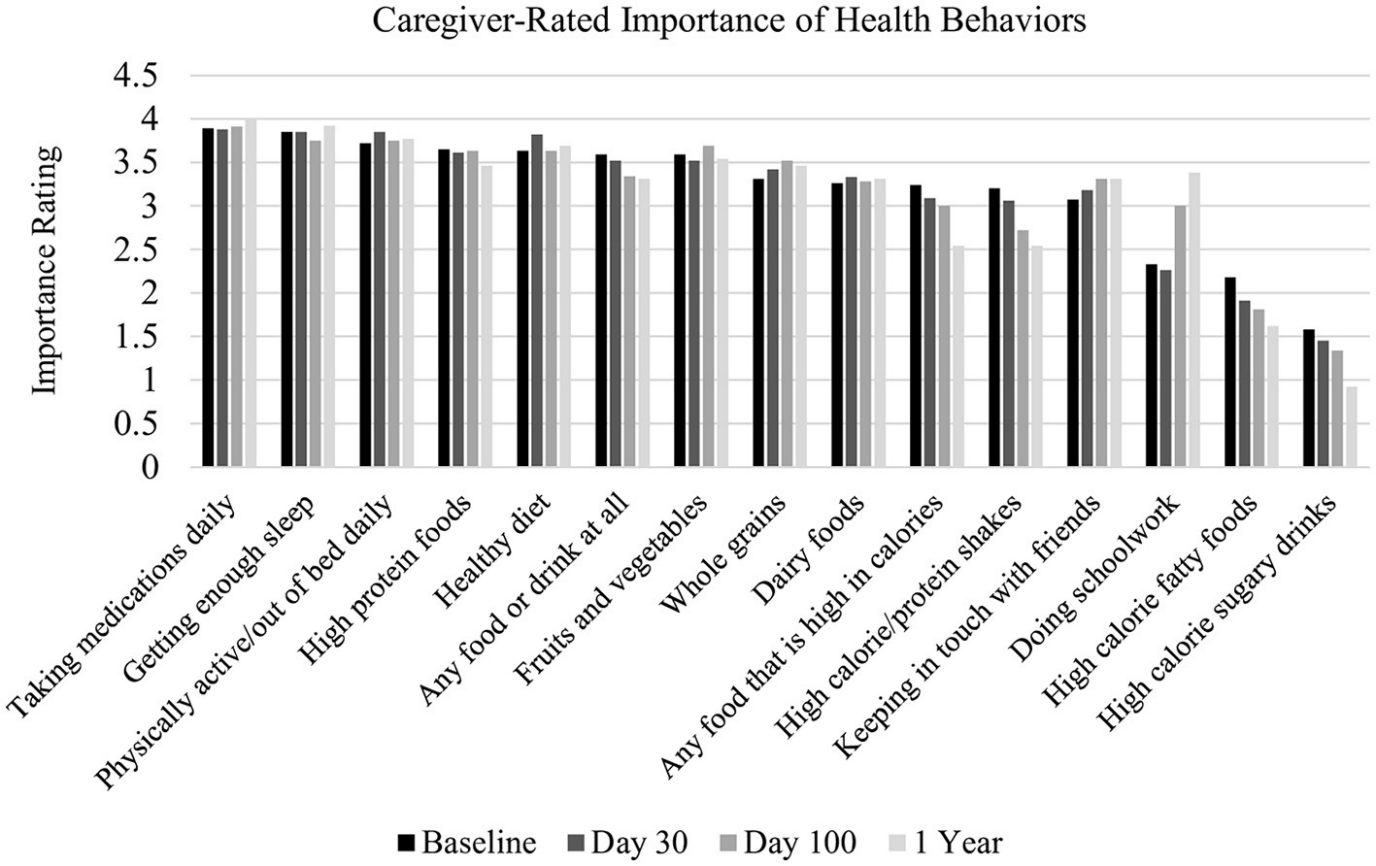
Caregiver-reported importance of health behaviors. X-axis label: 0 = not at all important, 1 = Unimportant, 2 = Somewhat important, 3 = Important, and 4 = Extremely important.

In the context of other health behaviors, caregivers rated eating a healthy diet as significantly more important for their child than keeping in touch with friends [*t*_(90)_ = 3.25, *p* < 0.01] and doing schoolwork [*t*_(90)_ = 5.53, *p* < 0.01] (see Figure 3). Caregivers rated eating a healthy diet as significantly less important than taking medications daily [*t*_(90)_ = −2.70, *p* < 0.01] and getting enough sleep [*t*_(90)_ = −2.21, *p* < 0.05]. Over half of caregivers reported that their child's illness has led to more emphasis on healthy eating (53%), and over one-quarter of caregivers reported that their child's illness did change parental dietary decisions (29%). A smaller percentage of caregivers reported that their child's illness led them to emphasize healthy eating less (13%).

#### 3.2.3 Confidence

Caregivers rated high confidence in their ability to provide healthy food for their child (Table 4). Overall, caregiver-rated confidence in their ability to provide healthy food did not significantly change across HSCT time points [*F*_(3, 118)_ = 1.63, *p* = 0.19]. At day 30 post-HSCT, confidence ratings were negatively correlated with caregiver depression and anxiety (*r* = −0.41, *p* < 0.05) and caregiver perceived stress (*r* = −0.42, *p* < 0.05), such that lower confidence in the ability to provide healthy food for their child was associated with higher caregiver anxiety/depression and stress. Confidence and caregiver stress were not significantly correlated pre-HSCT, at day 100 post-HSCT, or 1 year post-HSCT.

**Table 4:** Confidence and difficulty providing healthy food across time.

	**Time point**	**Mean**	**SD**	**Sig**.
Confidence	Pre-HSCT	2.84	0.68	
	Day 30	3.09	0.59	
	Day 100	3.10	0.69	
	1 Year	3.14	0.58	0.19
Difficulty	Pre-HSCT	1.10	1.06	
	Day 30	0.81	0.90	
	Day 100	0.81	0.95	
	1 Year	1.61	1.20	0.06

Sig = statistical significance of One-Way ANOVA comparing confidence and difficulty scores across time points. Higher scores = higher confidence in ability to provide healthy food and higher rated difficulty providing healthy food.

#### 3.2.4 Difficulty

**Figure 4:**
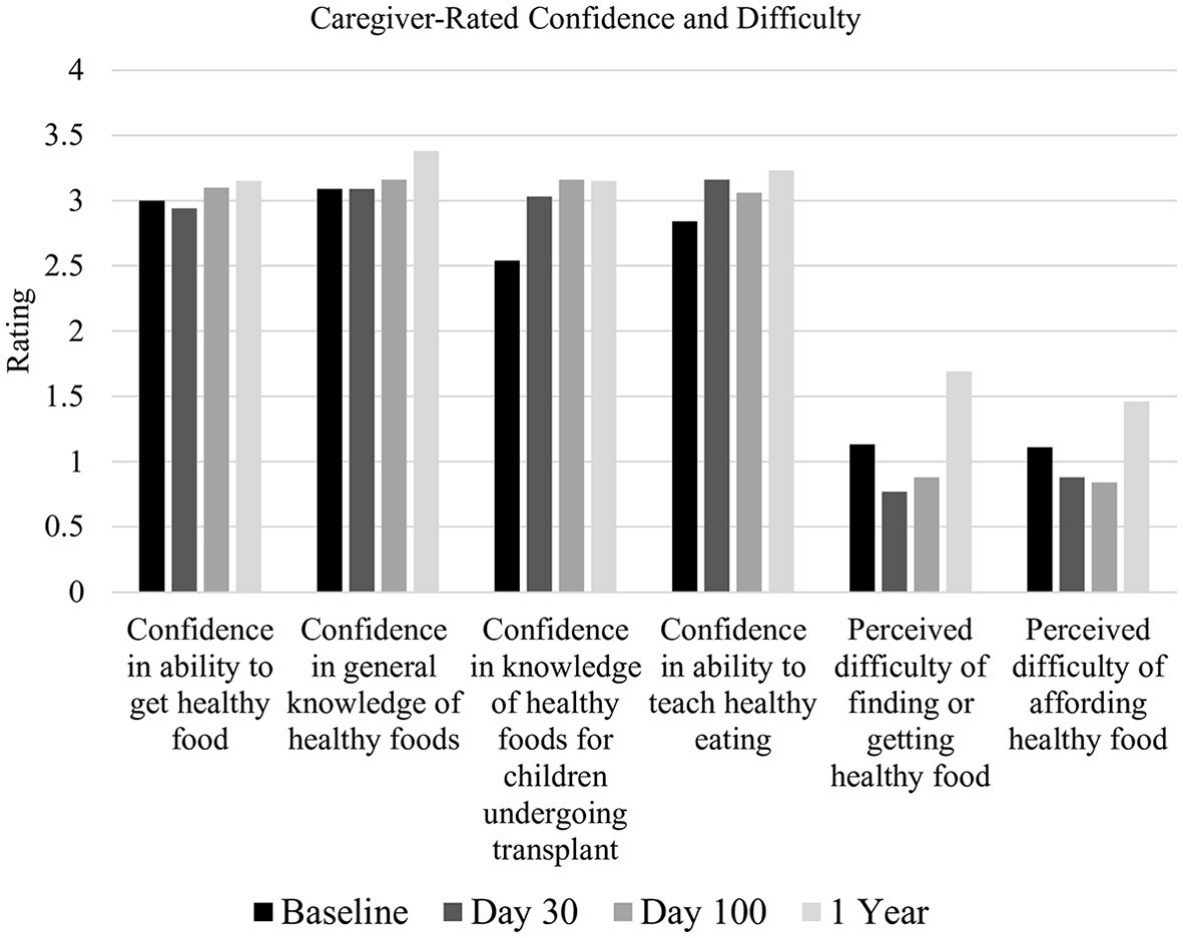
Caregiver-rated confidence and difficulty. X-axis: 0 = Not at all, 1 = A little, 2 = Somewhat, 3 = Very, and 4 = Extremely.

Caregivers generally rated low difficulty in providing healthy food for their child (Table 4). It did not significantly change across time points [*F*_(3, 118)_ = 2.57, *p* = 0.06] (see Figure 4).

Difficulty providing healthy food for their child was positively correlated with caregiver depression and anxiety pre-HSCT (*r* = 0.49, *p* < 0.001), 100 days post-HSCT (*r* = 0.57, *p* < 0.001), and 1 year post-HSCT (*r* = 0.74, *p* < 0.01), such that as caregiver distress increased, so did the difficulty to provide healthy food. At 30 days post-HSCT, difficulty providing healthy food was not significantly associated with caregiver depression and anxiety.

**Figure 5:**
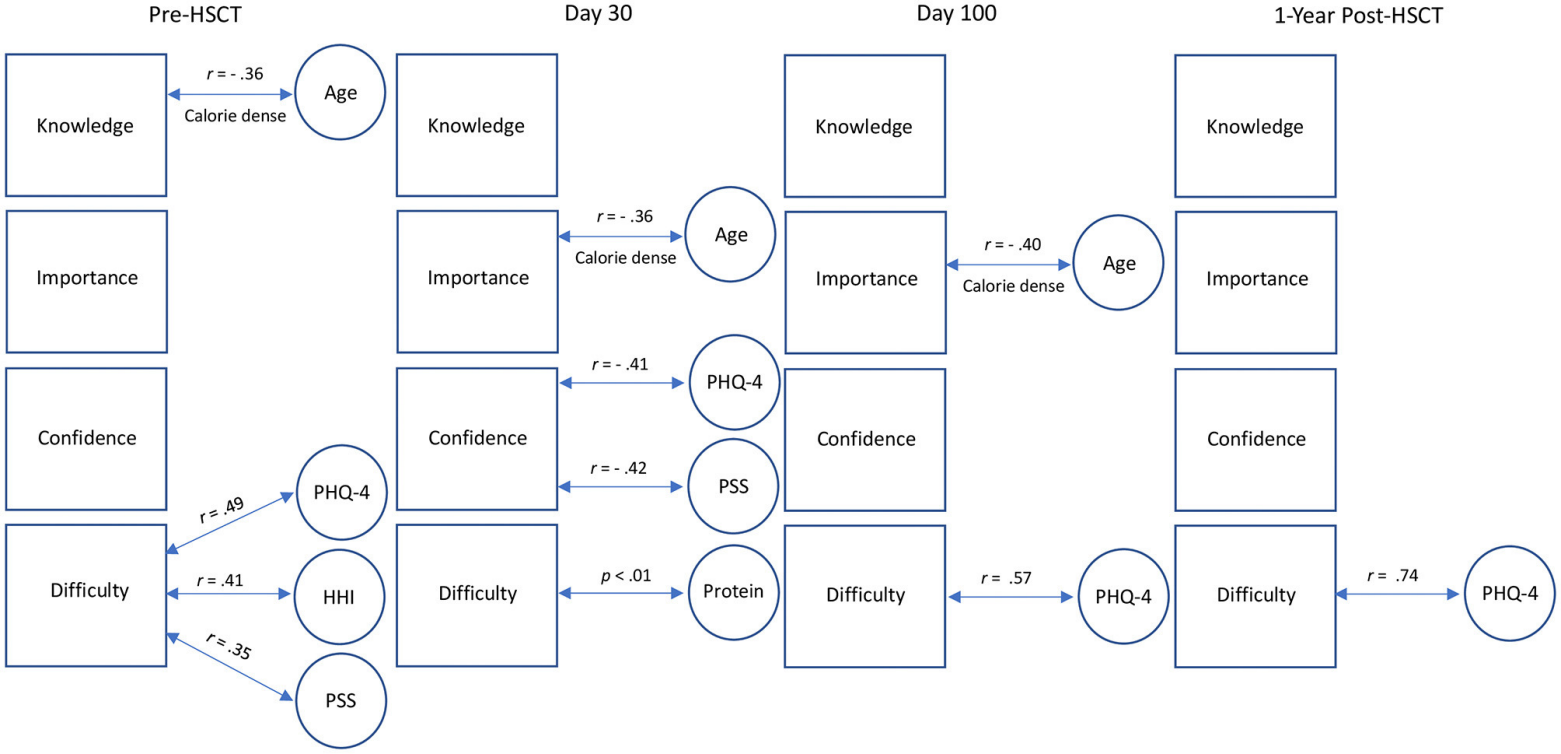
Visual depiction of dietary self-efficacy correlations. Squares represent dietary self-efficacy variables. Age = child age. PHQ-4, Patient Health Questionnaire completed by caregiver; PSS, Perceived Stress Scale completed by the caregiver; HHI, Help for Health Inventory completed by the caregiver.

Pre-HSCT, difficulty providing healthy food was significantly associated with annual household income (ρ = −0.8, *p* < 0.05), miscarried helping (*r* = 0.41, *p* < 0.01), and caregiver stress (*r* = 0.35, *p* < 0.05), such that lower income, higher miscarried helping, and greater caregiver stress were associated with more difficulty providing healthy food for their child. Post-HSCT, at 30 days, 100 days, and 1 year, difficulty providing healthy food was not significantly correlated with miscarried helping or caregiver stress. See Figure 5.

#### 3.2.5 Barriers

The most commonly reported barriers to following dietary guidelines were the child's dislike for healthy food, caregivers' desire that their child be happy and comfortable while they are ill, cost of healthy foods, child behavior interfering with healthy eating, other family members undermining caregiver efforts, and treatment side effects (e.g., nausea). See Table 5.

**Table 5:** Caregiver-reported barriers.

	**Baseline**		**Day 30**		**Day 100**		**1 Year**	
**Barriers**	***n***	**%**	***n***	**%**	***n***	**%**	***n***	**%**
My child doesn't like healthy food	16	(35)	9	(28)	9	(30)	4	(31)
I want my child to be happy/comfortable while ill	16	(35)	12	(36)	11	(34)	3	(23)
Healthy foods cost too much	12	(26)	9	(27)	7	(22)	4	(31)
My child's behavior interferes with eating healthy	11	(24)	6	(18)	6	(18)	1	(8)
Other family members undermine my efforts	9	(20)	4	(13)	5	(15)	2	(15)
My child is nauseated by the smell of food	7	(15)	8	(24)	4	(13)	0	(0)
My child has mouth sores	6	(13)	3	(9)	3	(9)	1	(8)
My way differs from other caregivers	6	(13)	3	(9)	3	(9)	3	(23)
Fast food is more convenient	6	(13)	4	(12)	5	(16)	1	(7)
My child is nauseous	6	(13)	5	(15)	5	(15)	2	(15)
I don't have time	6	(13)	0	(0)	4	(13)	0	(0)
My grocery store doesn't carry healthy options	2	(4)	1	(3)	0	(0)	0	(0)
My child has difficulty swallowing	2	(4)	3	(9)	3	(9)	0	(0)
Healthy meals take too long to prepare	1	(2)	2	(6)	0	(0)	0	(0)
The grocery store is too far away	1	(2)	0	(0)	0	(0)	0	(0)
I don't want to have to cook different for this child	1	(2)	0	(0)	0	(0)	0	(0)
Feeding junk food is my way of comforting	1	(2)	0	(0)	0	(0)	0	(0)
My child is worried about gaining weight	1	(2)	1	(3)	0	(0)	0	(0)
I don't know what are healthy foods	0	(0)	0	(0)	1	(3)	0	(0)
I am too stressed	0	(0)	0	(0)	1	(3)	0	(0)
I promise candy to get my child to behave	0	(0)	1	(3)	2	(6)	0	(0)
Healthy foods are not culturally traditional for my family	0	(0)	0	(0)	0	(0)	0	(0)

Pre-HSCT, caregivers were more likely to report the desire that their child be happy and comfortable while they are ill as a barrier to healthy eating if their child was younger (*r* = −0.42, *p* < 0.01). Caregivers of younger children were also more likely to report their child's behavior as a barrier to healthy eating (*r* = −0.37, *p* < 0.05). Caregivers who reported the cost of healthy food as a barrier reported lower household income (*r* = −0.49, *p* < 0.01). Caregivers who endorsed their child's dislike for healthy food reported greater anxiety and depression (*r* = 0.34, *p* < 0.05) and more miscarried helping (*r* = 0.47, *p* < 0.001). Caregivers who reported that other family members undermined their efforts reported greater depression and anxiety (*r* = 0.34, *p* < 0.05) and more caregiver stress (*r* = 0.39, *p* < 0.01). Post-HSCT, on day 30, caregivers who endorsed their desire for their child to be happy and comfortable while ill as a barrier to eating healthy reported greater depression and anxiety (*r* = 0.43, *p* < 0.05) and greater miscarried helping (*r* = 0.44, *p* < 0.05). On day 100 post-HSCT, caregivers who endorsed their child's dislike for healthy food reported greater anxiety and depression (*r* = 0.55, *p* < 0.01). In summary, caregiver depression and anxiety were associated with multiple perceived barriers at different time points.

### 3.3 Nutritional status

None of the pediatric patients met nutritional guidelines for fiber intake (Table 6). At no time point was fiber intake correlated with caregiver dietary self-efficacy (e.g., knowledge, importance, confidence, or difficulty), depression and anxiety, miscarried helping, or caregiver stress.

**Table 6:** Nutrition intake descriptive statistics.

		**Fiber**		**Protein**		**Added sugar**		**Saturated fat**	
	**Criteria**	**Met**	**Not met**	**Met**	**Not met**	**Met**	**Not met**	**Met**	**Not met**
Pre-HSCT	*N* (%)	0 (0)	20 (100)	13 (65)	7 (35)	15 (75)	5 (25)	15 (75)	5 (25)
	M (SD)	–	8.86 (4.91)	77.46 (40.34)	36.69 (8.01)	13.68 (12.58)	77 (38.72)	16.02 (4.29)	38.10 (7.60)
Day 30	*N* (%)	0 (0)	11 (100)	6 (55)	5 (45)	8 (73)	3 (27)	7 (64)	4 (36)
	M (SD)	–	9.59 (6.28)	89.78 (37.60)	20.70 (15.47)	24.37 (14.88)	82.85 (54.04)	12.35 (7.83)	36.03(14.03)
Day 100	*N* (%)	0 (0)	9 (100)	6 (66)	3 (33)	8 (89)	1 (11)	4 (44)	5 (56)
	M (SD)	–	11.37 (5.83)	97.43 (29.80)	44.06 (5.62)	21.66 (15.00)	47.33 (n/a)	16.74 (6.01)	33.56 (9.48)
1-Year	*N* (%)	0 (0)	4 (100)	3 (75)	1 (25)	3 (75)	1 (25)	3 (75)	1 (25)
	M (SD)	–	9.77 (4.22)	87.12 (22.14)	43.50 (n/a)	6.00 (4.67)	45.08 (n/a)	20.15 (4.76)	32.88 (n/a)

Values are expressed in grams. Recommended grams in fiber range from 14 to 28 depending on age and gender. Recommended protein ranges from 13 to 56 depending on age and gender. Recommended sugar is < 25 to 60 depending on age and gender. Recommended saturated fat is < 11.11 to 26.66 depending on age and gender.

At all-time points, caregiver ratings of healthiness and importance of nutrient-dense and calorie-dense foods and confidence to provide healthy food were statistically unrelated to whether the pediatric patient met protein, sugar, and saturated fat nutritional guidelines.

On day 30 post-HSCT, caregivers of pediatric patients who met nutritional guidelines for protein intake reported significantly less difficulty providing their child with healthy foods (M = 0.16, SD = 0.41) compared to caregivers of patients who did not meet nutritional guidelines for protein [M = 1.30, SD = 0.67; *t*_(9)_ = 3.46, *p* < 0.01]. However, this was not observed at pre-HSCT, day 100 post-HSCT, and 1 year post-HSCT. At no time point was caregiver-reported difficulty providing their child with healthy foods associated with meeting sugar or saturated fat nutritional guidelines.

Caregiver depression and anxiety, miscarried helping, and caregiver stress did not significantly differ between caregivers of pediatric patients who met or did not meet the nutrition guidelines for protein, sugar, and saturated fat.

### 3.4 Weight status

Pre-HSCT, 58% (*n* = 25) of pediatric patients fell within the healthy weight range, whereas 14% (*n* = 6) were underweight, and 28% (*n* = 12) were overweight (Table 7). At day 100 post-HSCT, results from a One-Way ANOVA showed significant differences in caregiver-reported difficulty providing healthy food for their child [*F*_(2, 28)_ = 4.34, *p* < 0.05]. Tukey post hoc multiple comparisons showed that caregivers of pediatric patients whose weight fell within the overweight range reported significantly more difficulty providing healthy food for their child (M = 3.17, SD = 2.22) compared to caregivers of pediatric patients whose weight fell within the healthy range (M = 0.88, SD = 1.27). Caregiver difficulty and pediatric weight status were not associated with pre-HSCT, 30 days post-HSCT, or 1 year post-HSCT.

**Table 7:** BMI Z-scores and BMI descriptive statistics.

		**BMI Z-score Age 2–19**						**BMI Age 20 and Older**					
		***n***	**%**	**Min**.	**Max**	***M***	***SD***	***n***	**%**	**Min**	**Max**	***M***	***SD***
Pre-HSCT (*N =* 43)	Underweight	6	(15)	−2.83	−1.14	−1.89	0.71	0	(0)				
	Healthy	23	(59)	−0.93	0.96	0.11	0.62	2	(50)	19.70	24.70	22.20	3.53
	Overweight	10	(26)	1.18	2.40	1.72	0.46	2	(50)	25.40	32.22	28.81	4.82
Day 30 (*n =* 31)	Underweight	5	(18)	−1.36	−2.64	−1.84	0.57	0	0				
	Healthy	14	(50)	−0.87	0.88	−0.08	0.63	2	(66)	19.70	23.87	21.79	2.95
	Overweight	9	(32)	1.01	2.09	1.43	0.38	1	(33)	25.40	25.40	25.40	n/a
Day 100 (*n =* 31)	Underweight	7	(25)	−2.73	−1.07	−1.65	0.58	0	(0)				
	Healthy	16	(57)	−0.78	0.88	−0.01	0.50	2	(66)	19.50	21.00	20.50	1.06
	Overweight	5	(18)	1.28	1.90	1.62	0.26	1	(33)	26.40	26.40	26.50	n/a
1 year (*n =* 14)	Underweight	2	(15)	−1.46	−1.06	−1.26	0.28	0	(0)				
	Healthy	8	(62)	−0.56	0.31	−0.13	0.32	0	(0)				
	Overweight	3	(23)	1.20	2.15	1.57	0.51	1	(100)	27.99	27.99	27.99	n/a

BMI Z-scores−1 and below were categorized as underweight. BMI scores 18.50 and below were categorized as underweight. BMI Z-scores between−1 and +1 were categorized as healthy. BMI scores between 18.5 and 25 were categorized as healthy. BMI Z-scores +1 and above were categorized as overweight. BMI scores 25 + were categorized as overweight. These categorizations were created based on Center for Disease Control (CDC). Percent are expressed as percentage of pediatric patients within each category for each age group. Total number of pediatric patients are lower than total number of pediatric patients enrolled in study due to one patient with no BMI and patients under two years of age.

At no time point were there statistically significant differences between caregivers of pediatric patients with underweight, healthy, or overweight status across the other caregiver dietary self-efficacy domains (i.e., healthiness, importance ratings of calorie-dense or nutrient-dense foods, or confidence).

Similarly, at no time point did caregiver depression, anxiety, miscarried helping, or stress significantly differ between caregivers of pediatric patients with underweight, healthy, or overweight BMI status.

## 4 Discussion

### 4.1 Key findings

As poor nutritional status has the potential to affect medical outcomes of pediatric HSCT patients (4), this study aimed to supplement the sparse literature on psychosocial factors affecting nutritional status, in particular the attitudes, mental health, and distress of the caregivers who are responsible for overseeing patients' nutritional intake. Our investigation of caregivers' self-efficacy in supporting child nutrition and its relevant emotional, behavioral, and transactional correlates produced a number of nuanced findings.

We found that caregivers endorsed the view that eating a healthy diet was important, albeit less important than other health-related behaviors, including taking medication and getting enough sleep, but more important than doing schoolwork and keeping up with friends. Caregivers demonstrated knowledge of what constituted healthy foods for children undergoing transplant. This finding suggests caregivers retained information given to them during dietary education efforts regarding children's healthy eating during HSCT. Our results do not show whether caregivers had pre-existing knowledge of healthy eating in general or whether their knowledge was a result of individually tailored guidance from a Registered Dietitian Nutritionist throughout the transplant process. However, dietary education is not the only factor that influences caregivers' self-reported efforts to provide nutritional support to pediatric patients.

Although caregivers consistently rated calorie-dense foods as relatively unhealthy across the transplant process, the perceived importance of such foods was greater earlier in the HSCT trajectory. For caregivers of younger children, the importance of calorie-dense food was rated significantly higher in the acute phase of HSCT, whereas caregivers reported calorie-dense foods as unhealthier for older children. This may be the result of parents' attempts to get their young child to eat “anything” compared to caregivers of older children, who may expect their adolescent or young adult child to make nutritious choices more autonomously. This inference is consistent with additional caregiver-reported barriers to their efforts to support healthy nutrition throughout the transplant process. The most commonly reported barriers, apart from the cost of healthy foods, were the child's dislike of healthy food, caregivers' desire that their child be happy and comfortable while they are ill, the child's behavior interfering with healthy eating, other family members undermining caregiver efforts, and the child experiencing nausea. Challenging behavior was more likely a barrier for caregivers of younger children.

Our findings, indicating that caregivers' well-intentioned efforts to provide nutritional support during transplant may have inadvertent negative effects among caregiver-pediatric patient dyads, add to the growing literature on miscarried helping. To date, miscarried helping has primarily been examined among caregivers and youth with chronic illnesses, such as diabetes and chronic pain. Interestingly, the average miscarried helping in this study was lower than that of published samples (24, 28). Further, in our HSCT sample, caregivers with greater depression and anxiety and higher miscarried helping were more likely to endorse that barriers to supporting their child in eating healthy foods were their desire that their child be happy and comfortable while they are ill and their child's dislike of healthy foods. Lastly, caregivers' perception that their efforts in supporting their child nutritionally were being undermined by other family members was also associated with greater caregiver depression and anxiety and higher caregiver stress. This finding suggests that dietary education for caregivers, as well as other family members who assist them, should be supplemented with psycho-education to mitigate caregiver stress and negative caregiver-child interactions surrounding eating.

The interplay of caregiver depression and anxiety with facets of self-efficacy supporting their child's nutrition warrants closer examination. During and immediately after the child's HSCT hospitalization (day 30), caregivers with more symptoms of depression and anxiety and higher stress reported less confidence in their ability to support their child in healthy nutrition. The acute phase is when the child is more likely to feel the sickest and less likely to want to eat or be able to do so, which may well explain the reduced caregiver confidence in supporting their child nutritionally. When the acute phase passed, the negative association between caregiver confidence, depression, anxiety, and stress dissipated.

Consistent with prior research finding associations between maternal depression and child food responsiveness in non-cancer samples, caregivers of children undergoing HSCT who reported more symptoms of depression and anxiety also endorsed greater difficulty providing their child with healthy food pre-HSCT, on day 100 post-HSCT, and 1 year post-HSCT. This association may not have been evident at day 30 post-HSCT as children are often still in the hospital where meals are provided. Pre-HSCT, caregivers with lower income, higher stress, and more negative parent-child interactions surrounding eating also reported greater difficulty providing healthy food; more difficulty providing healthy food was subsequently associated with overweight status at day 100 post-HSCT. During the most acute phase of the transplant trajectory, children of caregivers who reported greater difficulty providing healthy food were less likely to specifically meet protein nutritional guidelines, which may be related to the cost of nutritionally dense food vs. other less healthy foods and the high cost of high protein medical nutrition supplements. It may also be related to the child's preference for calorie-dense foods compared to protein-dense foods.

Despite overall high confidence and low difficulty in providing healthy food for their child, none of the pediatric patients met the fiber guidelines. Further, between 11% and 45% did not meet the nutritional guidelines for protein, sugar, or saturated fat. With the exception of difficulty providing healthy food at day 100 post-HSCT, dietary self-efficacy among caregivers, caregiver factors, and caregiver-child interactions were not associated with child BMI weight status in this small sample. However, our findings do tentatively suggest a potential avenue for improving adherence to healthy nutrition guidelines via identifying caregivers who themselves might be struggling emotionally, economically, or in their parenting relationship with their child and then directing specific resources to support caregivers of children undergoing HSCT.

### 4.2 Limitations

This study has several limitations. One is that the sample is relatively small and consequently has limited power to detect small but clinically meaningful associations. A second limitation is the homogeneity of the sample, which was primarily white and of relatively high socioeconomic status. Therefore, the barriers reported in this sample may not generalize to families with lower socioeconomic status. Additionally, our study did not collect aspects of social determinants of health other than income, which future studies with a more heterogeneous sample may want to consider (e.g., access to transportation and housing stability). A third limitation is that although the Dietary Self-Efficacy Scale was developed by a multidisciplinary team of hematology/oncology physicians, dieticians, and psychologists expert in the care of HSCT patients and specifically for the population of pediatric HSCT caregivers for whom the challenges in supporting their child nutritionally are unique and protracted in time, this scale could be enhanced by further validation in other treatment centers. Similarly, given the low internal reliability of miscarried helping in this sample compared to other published samples, the miscarried helping results should be interpreted with caution. Future research should specifically evaluate miscarried helping in the context of providing nutritional support in cancer populations, an important health-related behavior. A fourth limitation is that although BMI has been frequently used as a proxy for overall nutrition in many studies, BMI is less accurate early in the transplant trajectory when significant fluid shifts may occur. However, BMI is useful in tracking nutritional status over time and is interpreted in the context of the transplant trajectory to monitor health status. Lastly, as is common in longitudinal studies, particularly in medically vulnerable pediatric samples, this study experienced attrition, which increased with the passage of time. The food frequency questionnaire component appeared to be particularly challenging to participants at a time when the child was undergoing demanding medical treatment while medically very vulnerable. This high attrition limited longitudinal analyses.

### 4.3 Conclusions and future directions

Despite these limitations, this study's contributions to the literature rest on a fine-grained descriptive analysis of caregiver dietary self-efficacy and emotional, behavioral, and transactional facets of caregiver-child interactions in the context of caregiver efforts to provide nutritional support to their child undergoing HSCT. Caregivers were generally well-informed about the relative importance of nutrient-dense and calorie-dense foods but made exceptions for younger children. Overall, high caregiver confidence in supporting their child nutritionally was negatively affected by caregiver depression, anxiety, and stress only during or immediately after hospitalization. More difficulty providing such support at other time points was associated with lower income, higher stress, and more miscarried helping efforts. Future research with larger samples may cast more light on whether caregiver emotional, attitudinal, or dyadic caregiver-child transactional factors could affect child nutritional status as under- or over-nourished. The importance of this study lies in its congruence with a recently published narrative review of all aspects of nutritional support of pediatric HSCT patients that advocates for a multidisciplinary team to assess and tailor nutritional support for each patient (3). Our findings suggest that an important additional member of this team could be a psychosocial specialist whose contribution would be to assess and support the caregivers who may be challenged and struggling emotionally, behaviorally, or transactionally in caregiver-child interactions regarding nutrition and meeting nutritional guidelines.

## Data availability statement

The raw data supporting the conclusions of this article will be made available by the authors, without undue reservation.

## Ethics statement

This research study was approved by the University of Michigan Medical School Institutional Review Board (IRBMED). It was conducted in accordance with federal, state and institutional regulations. Participants aged 18 and over (caregivers and patients) provided written informed consent for their own participation. For patient participants who were minor children, caregivers (legal guardians/next of kin) provided informed consent.

## Author contributions

LL: Conceptualization, Data curation, Formal analysis, Methodology, Writing – original draft, Writing – review & editing. AN: Data curation, Formal analysis, Writing – original draft, Writing – review & editing. MB: Conceptualization, Writing – review & editing. FH: Conceptualization, Data curation, Formal analysis, Methodology, Supervision, Writing – original draft, Writing – review & editing. SB: Conceptualization, Writing – review & editing. SC: Conceptualization, Funding acquisition, Project administration, Resources, Writing – review & editing.
